# Characterization and phylogenetic analysis of a novel filamentous polymycovirus GbPmV1

**DOI:** 10.1002/mlf2.70046

**Published:** 2025-10-26

**Authors:** Hongjuan Bai, Linhao Song, Xin Luo, Weijie Chang, Jia Mi, Cheng Jin, Xiao Liu

**Affiliations:** ^1^ School of Clinical and Basic Medicine Shandong First Medical University & Shandong Academy of Medical Sciences Jinan China; ^2^ State Key Laboratory of Microbial Diversity and Innovative Utilization, Institute of Microbiology Chinese Academy of Sciences Beijing China; ^3^ College of Life Sciences University of Chinese Academy of Sciences Beijing China; ^4^ College of Veterinary Medicine Shanxi Agricultural University Jinzhong China; ^5^ College of Information Science and Technology Beijing University of Chemical Technology Beijing China

## Abstract

Mycoviruses are common in fungi and can change their host's functions. Here, we identify a novel dsRNA mycovirus GbPmV1 from the fungus *Gongronella butleri*. The genome of GbPmV1 exceeds 10,000 nucleotides and comprises six dsRNAs, with dsRNA1 encoding the RdRp and dsRNA6 encoding the capsid protein. GbPmV1 belongs to the family *Polymycoviridae* and shows unusual filamentous, virus‐like particles. Infection by GbPmV1 enhances the resistance of its fungal host to stresses and antifungal azoles. This study not only identifies a novel mycovirus in the zygomycete fungus *G. butleri* but also provides insights into the evolution and biological properties of polymycoviruses.

Mycoviruses are viruses that specifically infect fungi, replicating and reproducing within fungal cells^1^. Over the past few decades, research on mycoviruses has advanced rapidly, enhancing our understanding of viral diversity, taxonomy, and ecology^2^. According to the International Committee on Taxonomy of Viruses (ICTV), by March 2025, mycoviruses have been systematically classified into 48 families, 94 genera, and comprise 574 species. Their genomic types are predominantly double‐stranded RNA (dsRNA), positive single‐stranded RNA (+ssRNA), and negative single‐stranded RNA (‐ssRNA), with a smaller proportion being single‐stranded DNA^3^. Mycoviruses typically display symptomless infections, and do not exert significant pathological effects or pose existential threats to their hosts, which contrasts with other viruses^4^. However, a few mycoviruses can have either negative or positive impacts on the host^5^. Negative effects include reduced pathogenicity, impaired mycelial growth, disruption of toxin or pigment synthesis, and decreased spore production; these viruses are often termed hypovirulence‐associated mycoviruses. Conversely, specific mycoviruses can enhance the host's ability to adapt to environmental stress, as demonstrated by increased heat tolerance, enhanced pathogenicity, or promotion of certain toxin production^4^.

*Polymycoviridae* represents a newly established family as of 2020, characterized by their nontraditional capsid structures and relatively unique configurations^6^. The genome size of viruses in this family ranges from 7.5 to 12.5 kb. They contain at least four relatively conserved genomic segments, encoding the RNA‐dependent RNA polymerase (RdRp), a hypothesized scaffold protein involved in virion assembly, a methyltransferase, and a proline–alanine–serine‐rich protein (PASrp)^6^. Although most dsRNA viral particles display icosahedral structures, the *Polymycoviridae* family has two distinct structural forms: nonconventional virions and conventional filamentous capsids^6^. Based on the available genomic data and their unique structural domains, only one genus *Polymycovirus* has been identified within this family. Specifically, only four dsRNA viruses from this family have been described as possessing a filamentous capsid^7^, ^8^.

The genus *Gongronella*, a significant taxon within the fungal community, is primarily found in soil, plants, and various forms of humus, demonstrating strong growth capabilities and adaptability^9^. This genus plays a vital role in the ecosystem and has emerged as one of the most important microorganisms in commercial chitosan production, particularly represented by *Gongronella butleri* ^10^. As a member of the *Cunninghamellaceae* family within the *Zygomycota* phylum^10^, *G. butleri* is a soil‐dwelling fungus with notable applications in agriculture and medicine^9^. To date, no viruses have been reported in *G. butleri*.

In this study, we conducted dsRNA extraction to systematically screen fungal strains isolated from the rhizosphere of banana trees in Guangxi of China, aiming to identify potential mycoviruses^11^. Notably, we identified the strain 41‐5 containing dsRNAs after digestion with DNase I and S1 nuclease (Figure 1A). To characterize the genome structure of the dsRNA virus in strain 41‐5, we cloned the dsRNA sequences with random primers, and the dsRNA was ligated with the PC3‐T7 loop adaptor to obtain the 5′ and 3′ terminal sequences. Six viral nucleic acid sequences were identified, and the viral open reading frames (ORFs) were predicted using the NCBI ORF Finder. Remarkably, dsRNA1 was identified as a novel RdRp fragment of a polymycovirus, sharing 31.49% amino acid sequence identity (81% coverage) with *Botryosphaeria ramose* polymycovirus 1 (BrPmV1). Thus, the novel polymycovirus has been named *Gongronella butleri* polymycovirus 1 (GbPmV1). The genome of GbPmV1 comprises six dsRNA fragments of varying sizes, ranging from 1.2 kb to 2.8 kb, as schematically illustrated in Figure 1B.

**Figure 1:**
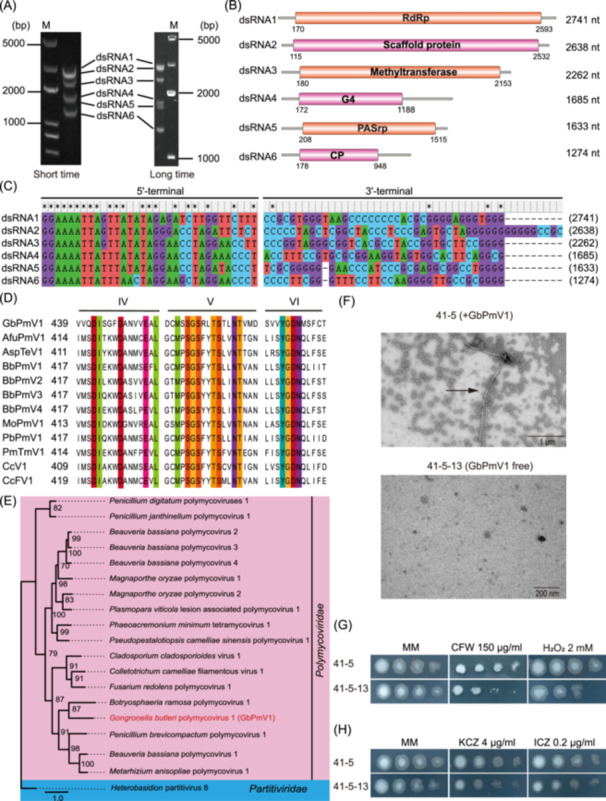
Characterization of the polymycovirus GbPmV1 and its impact on the growth of *Gongronella butleri* under various stress conditions. (A) Agarose gel electrophoresis of dsRNA from *G. butleri* 41‐5 stain digested by DNase I and S1 nuclease. The left panel shows a gel image from a short run, while the right panel displays a gel image from a long run. M, DNA marker. (B) Genome structure of the novel polymycovirus GbPmV1, with the ORFs highlighted as colored regions. (C) Multiple sequence alignment of the 5′‐terminal and 3ʹ‐terminal regions of the coding strands of the six dsRNA segments of GbPmV1. Conserved residues are indicated by asterisks. (D) Multiple alignment analysis based on the sequence of the conserved structural domain of the RdRp of GbPmV1 and 11 other members of the family *Polymycoviridae*. Conserved amino acid sites are marked by different colors. (E) A phylogenetic tree constructed based on the conserved structural domains of GbPmV1. The optimal model was identified as “Q.pfam+F+I+G4” by IQ‐TREE, and the number of bootstrap replicates was set to 1000. A presentative member of *Partitiviridae* was used as an outgroup. (F) Morphology of GbPmV1 viral particles in the 41‐5 strain by transmission electron microscopy. The virus‐free strain 41‐5‐13 is a negative control. Scale bar, 1 μm and 200 nm. (G) Colony morphology of virus‐infected strain 41‐5 and virus‐free strain 41‐5‐13 on MM medium supplemented with 150 µg/ml Calcofluor white (CFW) and 2 mM H_2_O_2_. (H) Colony morphology of virus‐infected strain 41‐5 and virus‐free strain 41‐5‐13 on MM medium supplemented with 4 µg/ml ketoconazole (KCZ) and 0.2 µg/ml itraconazole (ICZ). Concentrations of conidia were 1 × 10^7^, 1 × 10^6^, 1 × 10^5^, and 1 × 10^4^/ml in order.

Each dsRNA fragment contains an ORF accompanied by a long noncoding region that features conserved sequences at its terminus (Figure 1B,C). dsRNA1 contains a viral RdRp structural domain essential for viral replication, along with a 5′ untranslated region (UTR) of 170 nucleotides and a 3′ UTR of 148 nucleotides. dsRNA2 is proposed to function as a scaffold protein for viral particles, paired with a 115‐nucleotide 5′ UTR and a 106‐nucleotide 3′ UTR. dsRNA3 encodes a methyltransferase, accompanied by a 180‐nucleotide 5′ UTR and a 109‐nucleotide 3′ UTR. dsRNA4 encodes a protein with unknown functions, designated as G4. dsRNA5 contains a PASrp structural domain, with a 5′ UTR of 208 nucleotides and a 3′ UTR of 118 nucleotides. dsRNA6 encodes the capsid protein (CP) that encapsulates the virion nucleic acid, featuring a 5′ UTR of 178 nucleotides and a 3′ UTR of 326 nucleotides.

Previous studies have shown that terminal sequences of *Polymycoviridae* dsRNAs are often highly conserved. A comparative sequence alignment analysis of the 5′ and 3′ untranslated regions of the GbPmV1 viral genome reveals that both ends of dsRNAs 1 to 6 contain conserved sequences, with the 5′‐end nucleotide sequence being (GGAAAUUA) and the 3′‐end nucleotide ending with G/C^6^ (Figure 1C). Additionally, based on the alignment analysis of the RdRp of GbPmV1 and 11 other polymycoviruses, GbPmV1 shares three typical conserved motifs (IV–VI) with these mycoviruses (Figure 1D), indicating that GbPmV1 may be a member of the *Polymycoviridae* family.

To investigate the evolutionary relationship between GbPmV1 and other mycoviruses, we performed comparative analysis of its RdRp amino acid sequence with those of related viruses from the NCBI database. Phylogenetic reconstruction using IQ‐TREE revealed that GbPmV1 clusters with members of the *Polymycoviridae* family (Figure 1E). These results demonstrate that the mycovirus GbPmV1 infecting strain 41‐5 represents a novel member of the *Polymycovirus* genus within the *Polymycoviridae* family. Next, we also analyzed the distribution of polymycoviruses across diverse fungal host taxa (Figure S1). Although the most known polymycoviruses are found in fungi belonging to *Ascomycota*, the discovery of GbPmV1 in *G. butleri* indicates their presence in fungi belonging to *Zygomycota*, thus broadening the recognized host range of these viruses. This finding provides valuable insights for future studies on virus–host coevolution dynamics.

While most dsRNA viruses form spherical particles, members of the *Polymycoviridae* family, such as AfuPmV‐1 and BbPmV‐1, show unique structural organization, lacking conventional capsid proteins^6^. To characterize GbPmV1 from strain 41‐5, we first purified viral particles using 20%–50% sucrose density gradient centrifugation. Nucleic acid analysis revealed particle enrichment in the 30%–35% sucrose fraction (Figure S2A). SDS‐PAGE detected a single ~30 kDa structural protein (Figure S2B), consistent with the predicted mass of a GbPmV1‐encoded protein. To verify the results, we used mass spectrometry to identify the purified protein and confirmed that it corresponds to the capsid protein encoded by GbPmV1 dsRNA6 (Figure S3A,B). Furthermore, we used transmission electron microscopy to observe the viral particles, ultimately identifying filamentous, virus‐like particles over 3000 nm in length (Figure 1F). Notably, we detected some shorter particles (Figure S2C), likely representing fragmentation products of intact virions. Filamentous virus‐like particles remain uncommon in the *Polymycoviridae* family, although few filamentous virus‐like particles within the family have been identified since the first reported *Colletotrichum camelliae* filamentous virus 1 (CcFV‐1)^7^, ^8^. The discovery of GbPmV1 further confirmed that polymycoviruses may possess a unique encapsidation strategy, thereby enhancing our understanding of viral diversity in terms of structural characteristics. Notably, dsRNA viruses from the *Polyhedroviridae* family are phylogenetically closest to the +ssRNA‐associated Hadaka virus^12^. Our findings, together with previous studies^7^, ^8^, establish a foundation for investigating evolutionary transitions between ssRNA and dsRNA viral lineages.

Polymycovirus infections have been shown to induce morphological changes in the host, influence spore formation processes, and regulate growth rates and pathogenicity^13^, ^14^, ^15^, ^16^. CcFV‐1 and *Aspergillus fumigatus* polymycovirus 1 (AfPmV1) have been linked to decreased host growth rates or increased susceptibility to antifungal drugs^7^, ^15^. *Metarhizium anisopliae* polymycovirus 1 (MaPmV1) found in the insect pathogenic fungus *Streptomyces chlorostachybacterium* significantly enhanced host growth rates and conidial production, thereby improving its potential application in agricultural pest control^16^. To evaluate the impact of GbPmV1 on its fungal host, we attempted to eliminate GbPmV1 from virus‐infected strain 41‐5 by combining antiviral drug 2′‐C‐methylcytidine and single‐spore isolation (Figure S4A), which was subsequently confirmed through RT‐PCR (Figure S4B). The growth rate of strain 41‐5 was comparable to that of the virus‐free strain 41‐5‐13 (Figure S4C,D), indicating that GbPmV1 does not affect its host growth under normal conditions. Next, we investigated the effect of GbPmV1 on its host under stress conditions. We initially utilized calcofluor white (CFW), which primarily binds to chitin and cellulose in the fungal cell wall, disrupting normal biosynthesis and ultimately resulting in damage and dysfunction of the cell wall structure^17^. The strain carrying GbPmV1 showed enhanced resistance to the cell wall stress induced by CFW (Figure 1G). Additionally, H_2_O_2_ is a reactive oxygen species that attacks extracellular organic structures and penetrates the cell interior when it accumulates to a certain concentration, thereby inducing oxidative stress^18^. We examined the effect of GbPmV1 on the abiotic stress resistance of *G. butleri* by adding H_2_O_2_ to the MM medium to evaluate its antioxidant capacity. The results showed that under H_2_O_2_ stress, the strains carrying GbPmV1 showed stronger antioxidant capacity (Figure 1G), suggesting that GbPmV1 improves the tolerance of *G. butleri* to H_2_O_2_.

Previous studies have reported that the *Aspergillus fumigatus* tetramycovirus 1 virus (AfuTmV‐1), which belongs to the *Polymycoviridae* family like GbPmV1, infects *Aspergillus fumigatus* and increases its sensitivity to stress and antifungal drug nikkomycin Z^13^, ^14^. This prompted us to investigate the effect of GbPmV1 on sensitivity to antifungal drugs. Azoles are the most commonly used antifungal agents that inhibit the enzyme lanosterol demethylase to block the synthesis of ergosterol, thereby disrupting the fungal plasma membranes^19^. We selected two azole antifungals, ketoconazole (KCZ) and itraconazole (ICZ), for drug susceptibility testing. Interestingly, strain 41‐5 containing GbPmV1 showed enhanced resistance to both azoles in MM medium (Figure 1H). In addition, we analyzed the expression of H_2_O_2_ resistance genes (*cat1* and *cat3*) and drug resistance genes (*cdr4* and *erg11*) using RT‐qPCR. The GbPmV1‐infected strain 41‐5 showed higher mRNA levels of *cat3* compared to the virus‐free strain 41‐5‐13 (Figure S5A), consistent with its enhanced H₂O₂ resistance (Figure 1G). Similarly, the expression of *cdr4* and *erg11* was elevated in strain 41‐5 (Figure S5B), aligning with its increased ICZ resistance (Figure 1H). However, unlike the stress‐responsive genes, the chitosan biosynthesis genes (*chs1–chs3*) remained unaffected by GbPmV1 infection, despite the commercial relevance of *G. butleri* for chitosan production (Figure S5C). Together, these results suggest that GbPmV1 enhances the fungal host adaptation to various environmental stresses.

In conclusion, our study represents the identification of a novel dsRNA virus containing filamentous virus‐like particles in *G. butleri*. We characterized the complete nucleotide sequence, genome structure, and biological properties of GbPmV1, which is classified within the family *Polymycoviridae*. While polymycoviruses have been predominantly documented in ascomycetous fungi, the discovery of GbPmV1 in the zygomycete *G. butleri* significantly expands the known host range of the *Polymycoviridae* family, demonstrating its capacity to infect phylogenetically distant fungal lineages. Importantly, we identified structural features that are unusual for dsRNA viruses in this family through the observation of filamentous viral particles. Moreover, GbPmV1 enhances the resistance of *G. butleri* to cell wall stress and significantly reduces its susceptibility to azole antifungal drugs, suggesting that GbPmV1 may modulate the host's response mechanisms to external stresses by regulating its physiological state. These findings contribute to our understanding of the biological properties and evolution of mycoviruses and establish a foundation for future investigation into virus–host interaction mechanisms^4^, ^20^.

## AUTHOR CONTRIBUTIONS

**Hongjuan Bai**: Conceptualization; formal analysis; investigation; writing—original draft; writing—review and editing. **Linhao Song**: Formal analysis; investigation; writing—original draft; writing—review and editing. **Xin Luo**: Formal analysis; investigation. **Weijie Chang**: Formal analysis; investigation. **Jia Mi**: Formal analysis. **Cheng Jin**: Resources. **Xiao Liu**: Conceptualization; formal analysis; funding acquisition; resources; supervision; writing—original draft; writing—review and editing.

## ETHICS STATEMENT

This study did not involve any human participants or animal subjects.

## CONFLICT OF INTERESTS

The authors declare no conflict of interests.

## Supporting information

Supporting Information.

## Data Availability

All data generated or analyzed during this study are included in the manuscript and supporting files. Sequence data of dsRNAs 1–6 of GbPmV1 have been deposited in GenBank under accession numbers PV700488–PV700493, respectively. Materials are available from the corresponding author upon reasonable request.
