# Supplementary material for: Characterization and phylogenetic analysis of a novel filamentous polymycovirus GbPmV1
Source: mLife. 2025 Oct 26;4(5):572–6. doi: 10.1002/mlf2.70046 (PMC12575084; doi:10.1002/mlf2.70046)
Supplement: Supplementary file 1 — Supporting Information. [file MLF2-4-572-s001.docx]

**Supporting Information**

**Characterization and phylogenetic analysis of a novel filamentous polymycovirus GbPmV1**

Hongjuan Bai^1,2 #^, Linhao Song^2,3 #^, Xin Luo^2,3^, Weijie Chang^2,4^, Jia Mi^5^, Cheng Jin^2,3^, Xiao Liu^2,3^ *

1. School of Clinical and Basic Medicine, Shandong First Medical University & Shandong Academy of Medical Sciences, Jinan, Shandong 250117, China.

2. State Key Laboratory of Microbial Diversity and Innovative Utilization, Institute of Microbiology, Chinese Academy of Sciences, Beijing, 100101, China.

3. College of Life Sciences, University of Chinese Academy of Sciences, Beijing, 100049, China.

4. College of Veterinary Medicine, Shanxi Agricultural University, Jinzhong, 030801, China.

5. College of Information Science and Technology, Beijing University of Chemical Technology, Beijing, 100029, China.

# These authors contributed equally to this work.

* Corresponding author

To whom correspondence should be addressed Xiao Liu, Tel.: 86-010-64806107, Email: [liux@im.ac.cn](mailto:liux@im.ac.cn).

**Materials and Methods**

**Fungal isolation and culture conditions**

We collected rhizospheric soil samples from multiple banana cultivation sites across Guangxi, China, from which we isolated 130 fungal strains. Among these, strain 41-5 was selected for further study based on its infection with a novel polymycovirus. Species identification was confirmed as *Gongronella butleri* through PCR amplification (1) and sequencing of the internal transcribed spacer (ITS) region, followed by BLAST analysis. The virus-free strain 41-5-13 served as a control in subsequent experiments.

For biomass production, strains were grown on potato dextrose agar (PDA) medium overlaid with sterile cellophane for 5 days in the dark at 28°C prior to harvesting. To investigate the impact of mycovirus on host growth, we sampled the edges of the hyphae of strains using a 5 mm diameter punch and inoculated these samples onto 90 mm plates containing 20 mL of PDA. The plates were then incubated in the dark at 28°C. We assessed colony morphology and growth rates at 3-, 5-, and 7-days post-inoculation. The experiments were repeated three times.

**Extraction of dsRNAs of GbPmV1**

Fungal strains were cultured on PDA plates for 3–5 days at 28°C. Mycelia were harvested by surface scraping, immediately flash-frozen in liquid nitrogen, and pulverized to a fine powder using a pre-chilled mortar and pestle. The extraction of dsRNA was primarily performed using the cellulose chromatography method (2). The extracted dsRNA was treated with DNase I (TaKaRa, 2270A) and S1 nuclease (TaKaRa, 2410A) for 30 minutes at 37°C to eliminate DNA and ssRNA. Additionally, a 0.8% agarose gel was prepared to assess dsRNA quality while the remaining samples were used for terminal sequencing.

**Cloning of the genome sequences of GbPmV1**

Total RNA was isolated from strain 41-5 for viral genome characterization. First-strand cDNA synthesis was performed using the random primers 05RACE-3RT and M-MLV, followed by the sequential addition of 1 M NaOH, Tris-HCl and HCl for RNA degradation and cDNA denaturation. The single-stranded cDNA was purified using a PCR product recovery kit and used as a template for second-strand synthesis. The amplified cDNA products were ligated into a T-vector and transformed into *E. coli* for cloning. Positive recombinant clones were selected, sequenced, and the viral genome sequences were assembled and analyzed using DNAMAN.

The terminal sequences of all mycoviruses were obtained using the previously optimized amplification method (3). To facilitate rapid cDNA end amplification, 200-500 ng of total RNA was ligated with 1 µL of the ligated PC3-T7 Loop: 5'-GGATCCCGGGAATTCGGTAATACGACTCACTATATTTTTATAGTGAGTCGTATTA-3'. The reaction was first incubated at 70°C for 5 min and immediately chilled on ice for 5 min. RNA ligation was then performed using T4 RNA ligase (Beyotime, D7021) at 4°C for 16-24 hours. The ligated products were reverse transcribed with M-MLV reverse transcriptase to generate viral cDNA. For terminal sequence amplification, 1 μL cDNA served as template in a nested PCR approach: (1) Primary PCR with virus-specific primers F1/R1 and the PC2 adapter primer (5'-CCGAATTCCCGGGGGATCC-3'), followed by (2) Secondary PCR using 10-fold diluted primary products with nested primers F2/R2 and PC2. Target fragments (>200 bp) were gel-purified, cloned into pMD19-T vector (TaKaRa, 6013), and sequenced with M13F/M13R primers (BGI Genomics). Two biological replicates were performed, and the complete viral genome was assembled from consensus sequences. Primers for mapping the terminal sequences of dsRNA segments are listed in Table S1.

**Multiple alignment and phylogenetic analysis of GbPmV1 genome sequences**

To systematically analyze the amino acid sequences of mycovirus and elucidate its potential phylogenetic affinities (4), GbPmV1 was initially compared against the NCBI BLASTx database (http://blast.ncbi.nlm.nih.gov/Blast.cgi). The ORFs within the cDNA sequence of the mycovirus were predicted using the NCBI ORF Finder tool (https://www.ncbi.nlm.nih.gov/orffinder/). To predict the potential functions of the viral proteins, the Conserved Domain Database at NCBI was utilized (https://www.ncbi.nlm.nih.gov/Structure/cdd/wrpsb.cgi). A maximum likelihood (ML) phylogenetic tree was constructed using IQ-TREE software, version 1.6.11, with bootstrap values derived from 1000 replicates (5). The phylogenetic tree was visualized and edited using FigTree software, version 1.4.3, while Adobe Illustrator was employed for further visualization and aesthetic enhancement of the constructed phylogenetic tree. Accession numbers for all other viruses referenced in this work are listed in Table S2.

**Extraction and purification of mycoviral particles**

Viral particles from strain 41-5 were extracted using the methodology outlined previously (1, 6). Conidia of *G. butleri* were cultured on Petri dishes at 28°C for 7 days, after which approximately 40 g of virus-infected hyphae were harvested. The hyphal biomass was pulverized in liquid nitrogen and mixed with 0.05 M sodium phosphate buffer (8 mM Na₂HPO₄, 2 mM NaH₂PO₄, pH 7.4; 4 mL/g tissue) using sterile 50 mL centrifuge tubes. Cellular debris was removed through sequential centrifugation (1500 × g, 10 min to 8000 × g, 10 min; 4°C). The clarified lysate was ultracentrifuged (120,000 × g, 3 hours, 4°C) to pellet viral particles, which were then resuspended in 1 mL phosphate buffer and the mixture was allowed to dissolve overnight at 4°C. This crude extract was subsequently layered onto a 20%-50% sucrose gradient solution and centrifuged at 120000 × g for 6 hours at 4°C. At this stage, the bands located between milky white and light blue were carefully transferred using a syringe. The harvested fraction was concentrated by additional ultracentrifugation (120,000 × g, 2 hours, 4°C) and negatively stained with 1% uranyl acetate for transmission electron microscopy visualization of virion morphology.

**Elimination of the mycovirus from fungi**

To obtain virus-free isolates of strain 41-5, this study utilized a combination of single-spore isolation and treatment with the antiviral drug 2'-C-methylcytidine (7). Initially, a culture of *G. butleri* was grown on a PDA medium for five days, after which it was thoroughly washed with sterile water. The culture was vortex-mixed to create a conidial suspension, which was then filtered through specialized filter cloths to eliminate mycelium. The concentration of conidia was determined by counting the spores using a hemocytometer under a microscope. The final spore solution was diluted to a concentration of 1 × 10³/mL and subsequently added to 15 mL of PDA. In the lower layer of the 15 mL PDA medium, 30 µL of 2'-C-methylcytidine at a concentration of 0.2 mg/mL was introduced. The plate was then incubated at 28°C for 1 to 2 days. Finally, the strains were analyzed using RT-PCR to confirm the elimination of mycovirus. Primers used for verifying mycovirus by RT-qPCR were Forward: 5'-ATTCAGTGTGGAGGTCAGCC-3'; Reverse: 5'-TGTGGCGGAGGTTATGTACG-3'.

**Detection of the growth rate of *G. butleri* in response to various stresses and drugs**

The assay was performed using MM solid medium supplemented with the following stress agents: itraconazole (0.2 μg/mL), ketoconazole (4 μg/mL), H₂O₂ (2 mM), and calcofluor white (CFW; 150 μg/mL), with DMSO (1% v/v) as the control. Fresh conidia were harvested from 5-day-old cultures and resuspended in sterile distilled water. Serial dilutions were prepared to obtain spore concentrations ranging from 1 × 10⁴ to 1 × 10⁷ /mL. Aliquots (6 μL) of each dilution were spot-inoculated onto the drug-amended media. All plates were incubated at 28°C for 48 hours, after which colony growth was documented by digital photography.

**RNA extraction and RT-qPCR analysis**

Total RNA was isolated using TRIzol reagent (Invitrogen) followed by lithium chloride purification, as previously described with minor modifications (8, 9). Briefly, fungal tissues were homogenized in liquid nitrogen using a mortar and pestle, then lysed in ice-cold TRIzol reagent (containing 38% phenol, 0.8 M guanidine thiocyanate, 0.4 M ammonium thiocyanate, 0.1 M sodium acetate [pH 5.2], and 5% glycerol). Following a 5-minute incubation at room temperature, the chloroform was added (1:5, v/v) and the mixture was centrifuged at 12,000 × g for 15 min at 4°C. RNA was precipitated from the aqueous phase using 2.5 volumes of ethanol containing 10% sodium acetate (pH 5.2). The RNA pellet was then resuspended in nuclease-free water and further purified by adding 0.5 volumes of 7.5 M LiCl, followed by incubation at -20°C for 30 min. After centrifugation at 4°C, the purified RNA pellet was dissolved in RNase-free water and stored at -80°C until use.

RT-qPCR analysis was performed as previously described (10) with the following specifications. cDNA synthesis: 1 μg of total RNA was reverse transcribed using HiScript II reverse transcriptase. Quantitative PCR: amplification was performed using a CFX96 real-time PCR system with gene-specific primers targeting catalase genes: *cat1* and *cat3*, drug resistance genes: *cdr4* and *erg11*, chitin synthase genes: *chs1*, *chs2,* and *chs3*, and reference gene: β-tubulin (internal control). Primer sequences are provided in Table S1.

**References**

1. Yuan J, Qiao F, Chang W, Yang Y, Song L, Liu XL, et al. Application of a One-Step method for rapid detection of nucleic acids from fungi. Mycology. 2025:1-13.

2. Okada R, Kiyota E, Moriyama H, Fukuhara T, Natsuaki T. A simple and rapid method to purify viral dsRNA from plant and fungal tissue. J Gen Plant Pathol. 2015;81(2):103-7.

3. Potgieter AC, Page NA, Liebenberg J, Wright IM, Landt O, van Dijk AA. Improved strategies for sequence-independent amplification and sequencing of viral double-stranded RNA genomes. J Gen Virol. 2009;90:1423-32.

4. Tommaso P, Moretti S, Xenarios I, Orobitg M, Montanyola A, Chang JM, et al. T-Coffee: a web server for the multiple sequence alignment of protein and RNA sequences using structural information and homology extension. Nucleic Acids Res. 2011;39:W13-W7.

5. Nguyen LT, Schmidt HA, von Haeseler A, Minh BQ. IQ-TREE: A Fast and Effective Stochastic Algorithm for Estimating Maximum-Likelihood Phylogenies. Mol Biol Evol. 2015;32(1):268-74.

6. Yu X, Li B, Fu YP, Jiang DH, Ghabrial SA, Li GQ, et al. A geminivirus-related DNA mycovirus that confers hypovirulence to a plant pathogenic fungus. P Natl Acad Sci USA. 2010;107(18):8387-92.

7. Khan HA, Baig DI, Bhatti MF. An Overview of Mycoviral Curing Strategies Used in Evaluating Fungal Host Fitness. Mol Biotechnol. 2023;65(10):1547-64.

8. Liu XL, Yang Y, Hu Y, Wu J, Han C, Lu Q, et al. The nutrient-sensing GCN2 signaling pathway is essential for circadian clock function by regulating histone acetylation under amino acid starvation. Elife. 2023;12:e8524.

9. Lu Q, Yu M, Sun X, Zhou X, Zhang R, Zhang Y, et al. Circadian clock is critical for fungal pathogenesis by regulating zinc starvation response and secondary metabolism. Sci Adv. 2025;11(13):eads1341.

10. Yang Y, Duan Z, Liu XL, Li Z, Shen Z, Gong S, et al. Checkpoint kinases regulate the circadian clock after DNA damage by influencing chromatin dynamics. Nucleic Acids Res. 2025;53(5):gkaf162.

**Supplementary Figures**

**
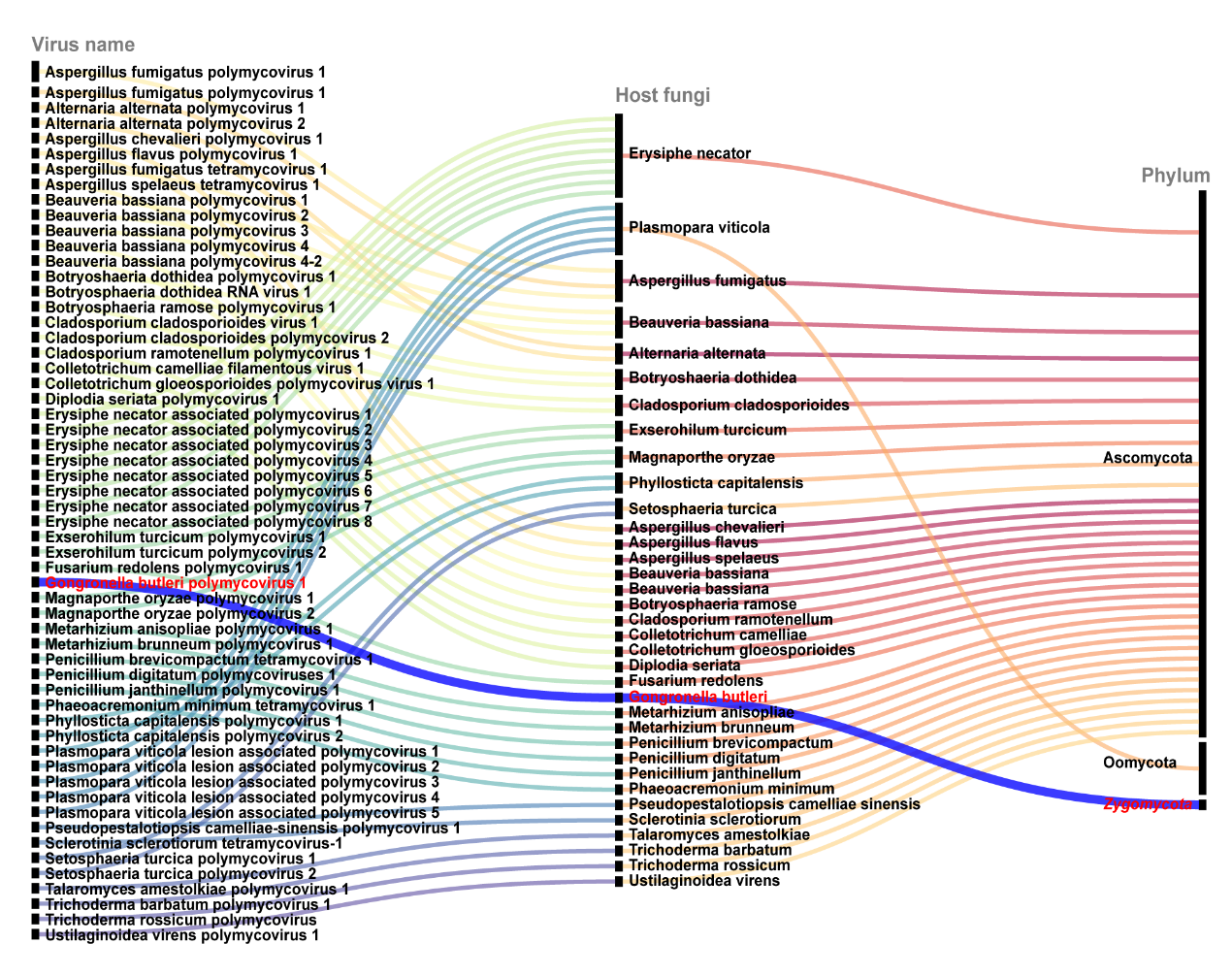
**

**Figure S1 Distribution of polymycoviruses across diverse fungal host taxa**

The Sankey diagram delineates the distribution of 58 fungal polymycoviruses among various fungal host taxa. GbPmV1 found in *G. butleri* indicates the presence of polymycovirus in Zygomycota.


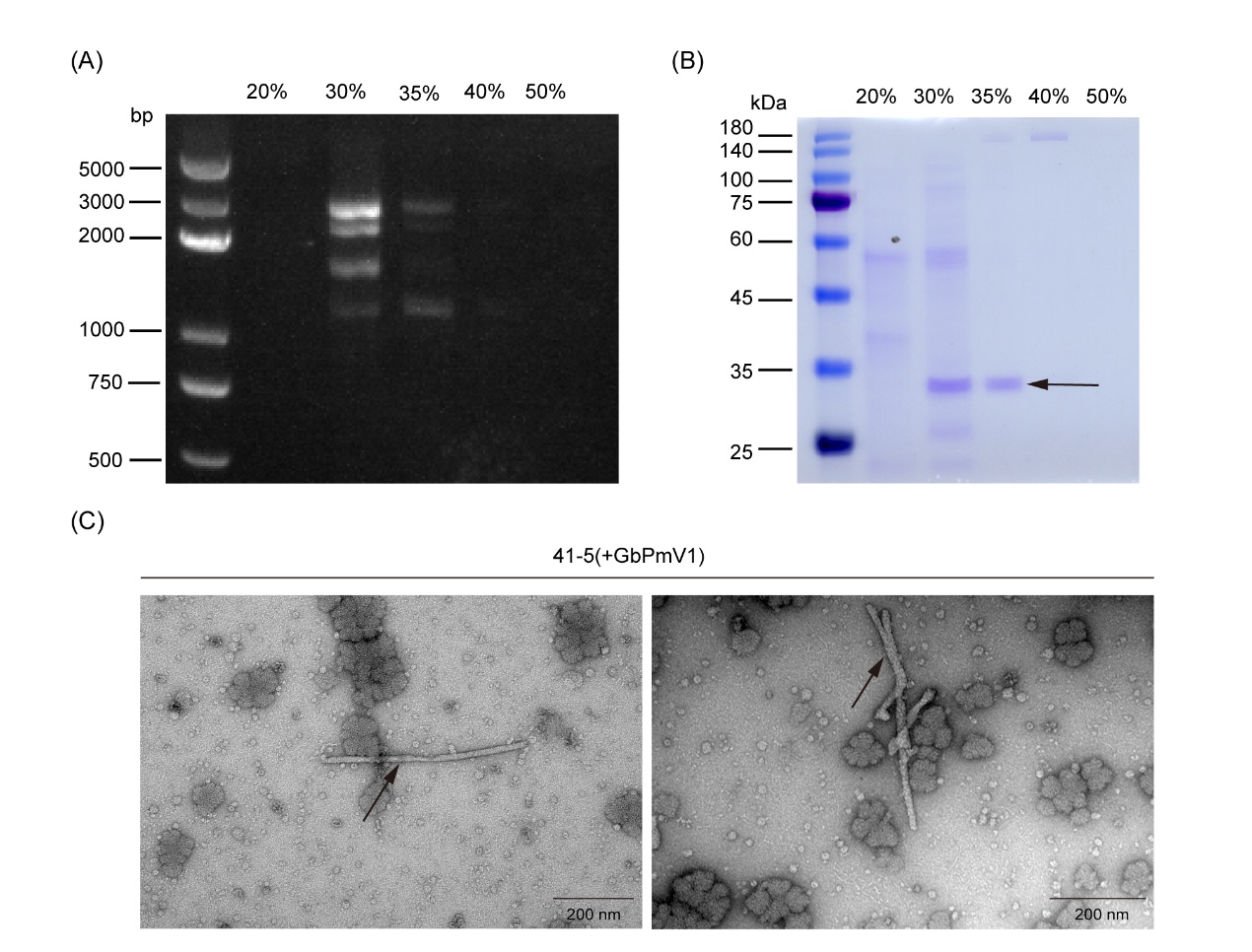


**Figure S2 Purification and detection of virus-like particles from strain 41-5 of *G. butleri***

(A) Agarose gel electrophoresis showing the distribution of dsRNAs of GbPmV1 in sucrose concentration gradient. (B) SDS-PAGE analysis of the distribution of viral proteins in sucrose concentration gradient. Gel was stained with Coomassie Blue. (C-D) Morphology of GbPmV1 polymycovirus by transmission electron microscopy from strain 41-5 of *G. butleri*. Scale bar, 200 nm.


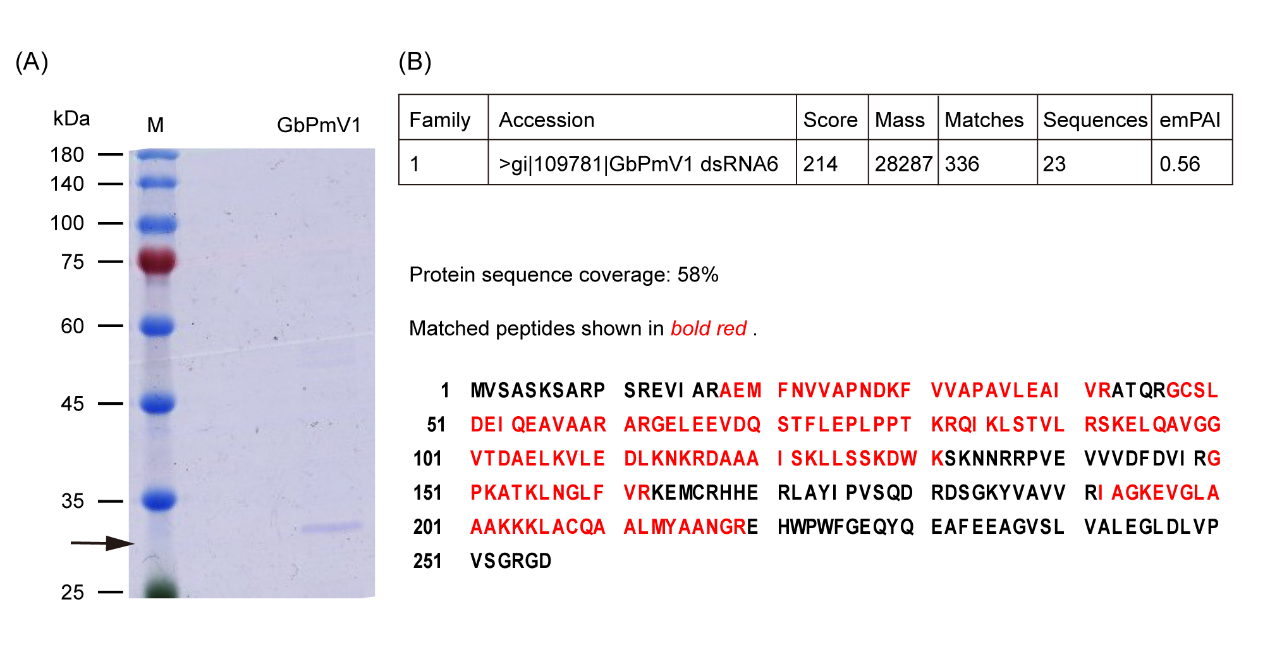


**Figure S3 Validation of the capsid protein by mass spectrometry**

(A) The peptide fragments derived from the GbPmV1 viral proteins in sucrose concentration gradient. (B) Mass spectrometry analysis showing the GbPmV1 capsid protein encoded by dsRNA6.


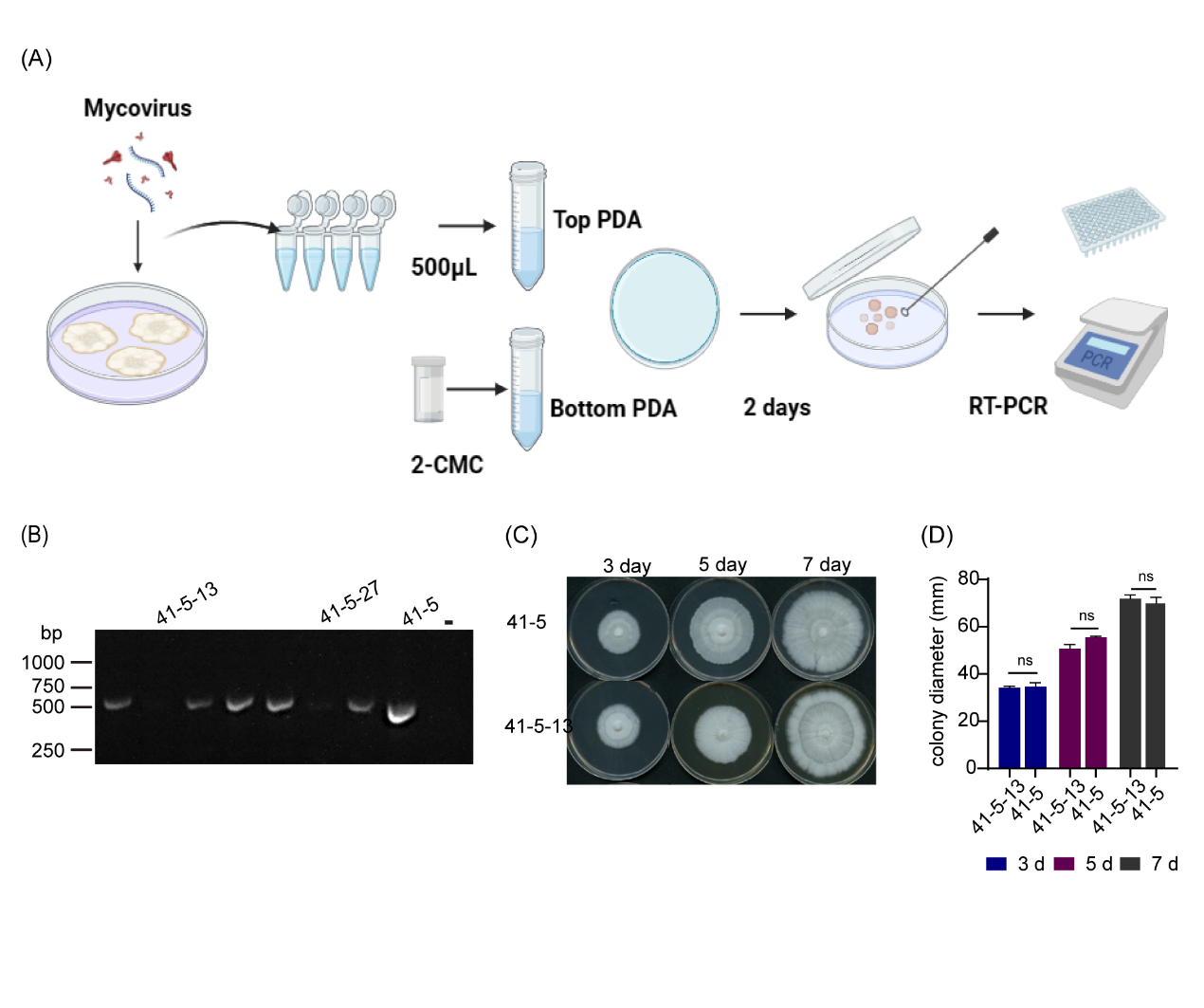


**Figure S4 Generation of a GbPmV1 virus-free strain**

(A) Schematic diagram illustrating the mycovirus elimination process using a combination of single-spore isolation and drug treatment. (B) Specific primers for GbPmV1 were designed to verify the presence or absence of GbPmV1 through RT-PCR. (C) Virus-infected strain 41-5 and virus-free strain 41-5-13 were cultured for 3-, 5-, and 7-days on PDA plates at 28°C in the dark, respectively. (D) Colony diameters were measured based on the growth of the virus-containing strain 41-5 and the virus-free strain 41-5-13.

**
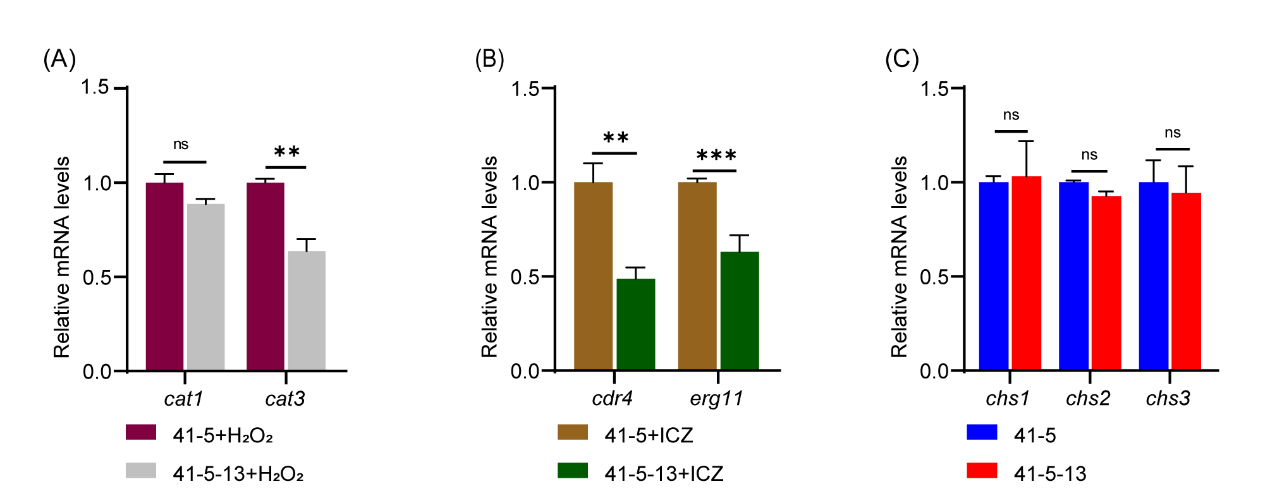
**

**Figure S5 Effect of GbPmV1 on gene expression of *G. butleri* under various stress conditions**

(A) RT-qPCR analysis showing the mRNA levels of *cat1* and *cat3* in the indicated strains in the presence of 5 mM H_2_O_2_. (B) RT-qPCR analysis showing the mRNA levels of *cdr4* and *erg11* in the indicated strains in the presence of 0.2 µg/mL itraconazole (ICZ). (C) RT-qPCR analysis showing the mRNA levels of *chs1, chs2* and *chs3* in the indicated strains. Bars represent mean ± SEM (n=3). Significance was assessed by using a two-tailed t-test. ** P<0.01, ***P< 0.001, ns P>0.05.

**Table S1. List of primers used in this study.**

| **Primers for RT-qPCR** | | |
| --- | --- | --- |
| *cat1* | *cat1* qPCR FP | ACGTCGCCAACACCTTGTAT |
|  | *cat1* qPCR RP | CAAGTCGGTCTTCTCCTCGG |
| *cat3* | *cat3* qPCR FP | TCCAACTACGCGCAAATCCT |
|  | *cat3* qPCR RP | AGAGTCTGCGAGTTTGGCTC |
| *chs1* | *chs1* qPCR FP | TGTTCGCACGCACGTTCCA |
|  | *chs1* qPCR RP | ACGACCGTCCGAGACAATGC |
| *chs2* | *chs2* qPCR FP | CGTGGTGAATGGCAAGCCTGT |
|  | *chs2* qPCR RP | ACAGCATCTGGCATGGCACAA |
| *chs3* | *chs3* qPCR FP | AAGGCAACCACGACGCTACA |
|  | *chs3* qPCR RP | TGATGCTGACACGGCTGAGA |
| *cdr4* | *cdr4* qPCR FP | TGACCGTTGCCGATGAGGAA |
|  | *cdr4* qPCR RP | GCCTGACAATGCCGCCAATG |
| *erg11* | *erg11* qPCR FP | CAAGGTGGTCGCCGACAAGAT |
|  | *erg11* qPCR RP | CGAGCCAGCGGTAAGGATTGT |
| *tubulin* | *tubulin* qPCR FP | AACGACCAATTCTCCGCCAT |
|  | *tubulin* qPCR RP | GGAGACGAGATCGTGCATGT |
| **Primers for mapping the terminal sequences of dsRNA segments** | | |
| dsRNA1 | dsRNA1F1 | TAGACAGCACTGCCTGGTTG |
|  | dsRNA1R1  dsRNA1F2  dsRNA1R2 | ACCATGGGGCTCCCATACAT  CAGTCGTGAGCGTATCCATC  CTGCCCTTGCCGGTAATCAT |
| dsRNA2 | dsRNA2F1 | GGAGAAGGGAGCGTCATC |
|  | dsRNA2R1  dsRNA2F2  dsRNA2R2 | CCTTGGGATAGGTCACTGG  CGAGTTCCCGTACACTCG  GGAGAGCGAGTTCTGAGC |
| dsRNA3 | dsRNA3F1  dsRNA3R1  dsRNA3F2  dsRNA3R2 | ACAAGACCAGGGGCAATAG  GAATACAAGGGGGGTATCTG  TACGCACCCATCGACAG  GGGATCGTTTTCTGCACTG |
| dsRNA4 | dsRNA4F1 | GCAACTTTCCCTCGTACAG |
|  | dsRNA4R1  dsRNA4F2  dsRNA4R2 | CAGGATGCAATCACACGTG  GGGAAGGGTTTCTAGGTTCC  GTCCTCAATCGTGGCTAGG |
| dsRNA5  dsRNA6 | dsRNA5F1  dsRNA5R1  dsRNA5F2  dsRNA5R2  dsRNA6F1  dsRNA6R1  dsRNA6F2  dsRNA6R2 | CAGATATTCGCGCCACTCTG  ATCCCAATCGATGCAGTCCC  CAAGTGCATTTGGGCCATAG  GATTACTAAGGAGGCTACCTGT  GTAGGCCACAACGGGTAGAC  ACGACGTTGAACATCTCGGC  CGTCGTTCTACCTACAGTGG  TGCAAAATTTAAACGAGTTGGG |

**Table S2 Accession numbers for all other viruses referenced in this work.**

| **Number** | **Virus name** | **GenBank accession numbers** |
| --- | --- | --- |
| 1 | Alternaria alternata polymycovirus 1 | dsRNA 1: MT345016; dsRNA 2: MT345017; dsRNA 3: MT345018; dsRNA 4: MT345019; dsRNA 5: MT345020; dsRNA 6: MT345021; dsRNA 7: MT345022; dsRNA 8: MT345023 |
| 2 | Alternaria alternata polymycovirus 2 | dsRNA1: OQ054001; dsRNA2: OQ054002; dsRNA3: OQ054003; dsRNA4: OQ054004; dsRNA5: OQ054005; dsRNA2: OQ054006; dsRNA2: OQ054007 |
| 3 | Aspergillus chevalieri polymycovirus 1 | dsRNA1: LC832452; dsRNA2: LC832453; dsRNA3: LC832454; dsRNA4: LC832455 |
| 4 | Aspergillus flavus polymycovirus 1 | dsRNA1: MZ600062; dsRNA2: MZ600063; dsRNA3: MZ600064; dsRNA4: MZ600065 |
| 5 | Aspergillus fumigatus polymycovirus 1 | dsRNA 1: LC553675; dsRNA 2: LC553676; dsRNA 3: LC553677; dsRNA 4: LC553678 |
| 6 | Aspergillus fumigatus polymycovirus 1 | dsRNA1: MH192993; dsRNA2: MH192994; dsRNA3: MH192995; dsRNA4: MH192996 |
| 7 | Aspergillus fumigatus polymycovirus 1 | dsRNA1: LC517041; dsRNA2: LC517042; dsRNA3: LC517043; dsRNA4: LC517045; dsRNA5: LC517044 |
| 8 | Aspergillus fumigatus tetramycovirus 1 | dsRNA1: HG975302; dsRNA2: HG975303; dsRNA3: HG975304; dsRNA4: HG975305 |
| 9 | Aspergillus spelaeus tetramycovirus 1 | dsRNA1: MG887754; dsRNA2: MG887755; dsRNA3: MG887756; dsRNA4: MG887757 |
| 10 | Beauveria bassiana polymycovirus 1 | dsRNA1: LN896307; dsRNA2: LN896308; dsRNA3: LN896309; dsRNA4: LN896310 |
| 11 | Beauveria bassiana polymycovirus 2 | dsRNA 1: LN896311; dsRNA 6: LN896312; dsRNA 7: LN896313 |
| 12 | Beauveria bassiana polymycovirus 3 | dsRNA 1: LR991938; dsRNA 2: LR991939; dsRNA 3: LR991940; dsRNA 4: LR991941; dsRNA 5: LR991942; dsRNA 6: LR991943 |
| 13 | Beauveria bassiana polymycovirus 4 | dsRNA 1: MW385785; dsRNA 2: MW385786; dsRNA 3: MW385787; dsRNA 4: MW385788; dsRNA 5: MW385789; dsRNA 6: MW385790 |
| 14 | Beauveria bassiana polymycovirus 4-2 | NON |
| 15 | Botryoshaeria dothidea polymycovirus 1 | dsRNA1: KT372135; dsRNA2: KT372136; dsRNA3: KT372137; dsRNA4: KT372138; dsRNA5: KT372139 |
| 16 | Botryosphaeria dothidea RNA virus 1 | dsRNA1: KP245734; dsRNA2: KP245735; dsRNA3: KP245736; dsRNA4: KP245737; dsRNA5: KP245738 |
| 17 | Botryosphaeria ramose polymycovirus 1 | NON |
| 18 | Cladosporium cladosporioides virus 1 | dsRNA1: KJ787686; dsRNA2: KJ787687; dsRNA3: KJ787688; dsRNA4: KJ787689; dsRNA5: KJ787690 |
| 19 | Cladosporium cladosporioides polymycovirus 2 | dsRNA1: OQ053989; dsRNA2: OQ053990; dsRNA3: OQ053991; dsRNA4: OQ053992; dsRNA5: OQ053993; dsRNA6: OQ053994 |
| 20 | Cladosporium ramotenellum polymycovirus 1 | dsRNA1: OQ053977; dsRNA2: OQ053978; dsRNA3: OQ053979; dsRNA4: OQ053980; dsRNA5: OQ053981; dsRNA6: OQ053982 |
| 21 | Colletotrichum camelliae filamentous virus 1 | dsRNA1: KX778766; dsRNA2: KX778767; dsRNA3: KX778768; dsRNA4: KX778769; dsRNA5: KX778770; dsRNA6: KX778771; dsRNA7: KX778772; dsRNA8: KX778773 |
| 22 | Colletotrichum gloeosporioides polymycovirus virus 1 | dsRNA1: OM937850; dsRNA2: OM937851; dsRNA3: OM937852; dsRNA4: OM937853; dsRNA5: OM937854; dsRNA6: OM937855; dsRNA7: OM937856; dsRNA8: OM937857; dsRNA9: OM937858 |
| 23 | Diplodia seriata polymycovirus 1 | dsRNA1: OM837794; dsRNA2: OM837795; dsRNA3: OM837796; dsRNA4: OM837797 |
| 24 | Erysiphe necator associated polymycovirus 1 | dsRNA1: MN617797; dsRNA2: MN617798; dsRNA3: MN617799 |
| 25 | Erysiphe necator associated polymycovirus 2 | dsRNA1: MN617800; dsRNA2: MN617801; dsRNA3: MN617802; dsRNA4: MN617803 |
| 26 | Erysiphe necator associated polymycovirus 3 | dsRNA1: MN617804; dsRNA2: MN617805; dsRNA3: MN617806; dsRNA4: MN617807 |
| 27 | Erysiphe necator associated polymycovirus 4 | dsRNA1: MN617808; dsRNA2: MN617809; dsRNA3: MN617810 |
| 28 | Erysiphe necator associated polymycovirus 5 | dsRNA1: MN617811; dsRNA2: MN617812; dsRNA3: MN617813; dsRNA4: MN617814 |
| 29 | Erysiphe necator associated polymycovirus 6 | dsRNA1: MN617815; dsRNA2: MN617816; dsRNA3: MN617817 |
| 30 | Erysiphe necator associated polymycovirus 7 | dsRNA1: MN617818 |
| 31 | Erysiphe necator associated polymycovirus 8 | dsRNA1: MN617819; dsRNA2: MN617820 |
| 32 | Exserohilum turcicum polymycovirus 1 | dsRNA1: PP926255; dsRNA2: PP926256; dsRNA3: PP926257; dsRNA4: PP926258; dsRNA5: PP926259 |
| 33 | Exserohilum turcicum polymycovirus 2 | dsRNA1: PP926250; dsRNA2: PP926251; dsRNA3: PP926252; dsRNA4: PP926253; dsRNA5: PP926254 |
| 34 | Fusarium redolens polymycovirus 1 | dsRNA1: MK609920; dsRNA2: MK609921; dsRNA3: MK609922; dsRNA4: MK609923; dsRNA5: MK609924; dsRNA6: MK609925; dsRNA7: MK609926; dsRNA8: MK609927 |
| 35 | Gongronella butleri polymycovirus 1 | dsRNA1: PV700488; dsRNA2: PV700489; dsRNA3: PV700493; dsRNA4: PV700490; dsRNA5: PV700491; dsRNA6: PV700492 |
| 36 | Magnaporthe oryzae polymycovirus 1 | dsRNA1: NC_055282; dsRNA2: NC_055283; dsRNA3: NC_055284; dsRNA4: NC_055285 |
| 37 | Magnaporthe oryzae polymycovirus 2 | dsRNA1: MW752168; dsRNA2: MW752169; dsRNA3: MW752170; dsRNA4: MW752171 |
| 38 | Metarhizium anisopliae polymycovirus 1 | dsRNA1: OP627094; dsRNA2: OP627095; dsRNA3: OP627096; dsRNA4: OP627097 |
| 39 | Metarhizium brunneum polymycovirus 1 | dsRNA1: OP524132; dsRNA2: OP524133; dsRNA3: OP524134; dsRNA4: OP524135 |
| 40 | Penicillium brevicompactum tetramycovirus 1 | dsRNA1: MG887750; dsRNA2: MG887751; dsRNA3: MG887752; dsRNA4: MG887753 |
| 41 | Penicillium digitatum polymycoviruses 1 | dsRNA1: MF317878; dsRNA2: MF317879; dsRNA3: MF317880; dsRNA4: MF317881; dsRNA5: NC_040422; dsRNA6: NC_040423; dsRNA7: NC_040424; dsRNA8: NC_040425 |
| 42 | Penicillium janthinellum polymycovirus 1 | dsRNA 1: LC571078; dsRNA 2: LC571079; dsRNA 3: LC571080; dsRNA 4: LC571081; dsRNA 5: LC571082 |
| 43 | Phaeoacremonium minimum tetramycovirus 1 | dsRNA1: MK584824; dsRNA2: MK584825; dsRNA3: MK584826; dsRNA4: MK584827 |
| 44 | Phyllosticta capitalensis polymycovirus 1 | dsRNA1: PP359411; dsRNA2: PP359412; dsRNA3: PP359413; dsRNA4: PP359414; dsRNA5: PP359415 |
| 45 | Phyllosticta capitalensis polymycovirus 2 | dsRNA1: PP359416; dsRNA2: PP359417; dsRNA3: PP359418; dsRNA4: PP359419; dsRNA5: PP359420 |
| 46 | Plasmopara viticola lesion associated polymycovirus 1 | dsRNA 1: MN557029; dsRNA 2: MN557030 |
| 47 | Plasmopara viticola lesion associated polymycovirus 2 | dsRNA 1: MN557032; dsRNA 2: MN557031; dsRNA 4: MN557033 |
| 48 | Plasmopara viticola lesion associated polymycovirus 3 | dsRNA 1: MN557034 |
| 49 | Plasmopara viticola lesion associated polymycovirus 4 | dsRNA 1: MN557035 |
| 50 | Plasmopara viticola lesion associated polymycovirus 5 | dsRNA 1: MN557036; dsRNA 2: MN557037; dsRNA 4: MN557038 |
| 51 | Pseudopestalotiopsis camelliae-sinensis polymycovirus 1 | dsRNA1: PP359405; dsRNA2: PP359406; dsRNA3: PP359407; dsRNA4: PP359408; dsRNA5: PP359409; dsRNA6: PP359410 |
| 52 | Sclerotinia sclerotiorum tetramycovirus-1 | dsRNA 1: MF444217; dsRNA 2: MF444218; dsRNA 3: MF444219 |
| 53 | Setosphaeria turcica polymycovirus 1 | dsRNA1: MW429374; dsRNA2: MW429375; dsRNA3: MW429376; dsRNA4: MW429377; dsRNA5: MW429378 |
| 54 | Setosphaeria turcica polymycovirus 2 | dsRNA1: OQ433941; dsRNA2: OQ433942; dsRNA3: OQ433943; dsRNA4: OQ433944; dsRNA5: OQ433945 |
| 55 | Talaromyces amestolkiae polymycovirus 1 | dsRNA1: OP096450; dsRNA2: OP096451; dsRNA3: OP096452; dsRNA4: OP096453; dsRNA5: OP096454; dsRNA6: OP096455 |
| 56 | Trichoderma barbatum polymycovirus 1 | dsRNA1: OM307406; dsRNA2: OM307407; dsRNA3: OM307408; dsRNA4: OM307409 |
| 57 | Trichoderma rossicum polymycovirus | NON |
| 58 | Ustilaginoidea virens polymycovirus 1 | dsRNA1: PP130091; dsRNA2: PP130092; dsRNA3: PP130093; dsRNA4: PP130094; dsRNA5: ON791679 |
| 59 | Heterobasidion partitivirus 8 | dsRNA1: JX625227; dsRNA2: JX625228 |
